# Formulation and evaluation of chitosan solid lipid nanoparticles of carbamazepine

**DOI:** 10.1186/1476-511X-11-72

**Published:** 2012-06-13

**Authors:** Rahul Nair, Ashok CK Kumar, Vishnu K Priya, Chakrapani M Yadav, Prasanna Y Raju

**Affiliations:** 1 Department of Pharmaceutics, Sree Vidyanikethan College of Pharmacy, Sree Sainath Nagar, Tirupati-517102, Andhra Pradesh, India

**Keywords:** Solid lipid nanoparticles, Chitosan, Carbamazepine, Encapsulation efficiency

## Abstract

The present work aims at preparing aqueous suspension of Solid lipid Nanoparticles containing Chitosan (CT) which is a biopolymer that exhibits a number of interesting properties which include controlled drug delivery. Carbamezapine (CBZ) is a lipophilic drug which shows it antiepileptic activity by inactivating sodium channels. The solid lipid Nanoparticles (SLN) of Chitosan-CBZ were prepared by using solvent injection method using ethanol as organic solvent. The prepared SLN formulations exhibited high encapsulation efficiency, high physical stability. The drug incorporated SLNs have demonstrated that the controlled release patterns of the drug for prolonged period. The prepared SLNs were characterized for surface morphology by SEM analysis, entrapment efficiency, zeta potential, FTIR, DSC and *In-vitro* diffusion studies. The hydrodynamic mean diameter and zeta potential were 168.7 ±1.8 nm and −28.9 ±2.0 mV for SLN-chitosan-CBZ respectively. Therefore chitosan-SLN can be good candidates to encapsulate CBZ and to increase its therapeutic efficacy in the treatment of Epilepsy.

## Introduction

Solid Lipid Nanoparticles (SLN) mainly comprise lipids that are in solid phase at the room temperature and surfactants for emulsification, the mean diameters of which range from 50 to 1000nm for colloid drug delivery applications [1]. SLNs offer unique properties such as small size, large surface area, high drug loading, the interaction of phases at the interfaces, and are attractive for their potential to improve performance of pharmaceuticals, neutraceuticals and other materials [2]. The typical methods of preparing SLNs include spray drying [3], high shear mixing [4], ultra-sonication [5,6], and high pressure homogenization (HPH) [7,8]. Advantages of SLN are the use of physiological lipids, the avoidance of organic solvents in the preparation process, and a wide potential application spectrum (dermal, oral and intravenous). Additionally, improved bioavailability, protection of sensitive drug molecules from the environment (water, light) and controlled and/or targeted drug release [9-11], improved stability of pharmaceuticals, feasibilities of carrying both lipophilic and hydrophilic drugs and most lipids being biodegradable [12,13]. SLNs possess a better stability and ease of upgradability to production scale as compared to liposomes. This property may be very important for many modes of targeting. SLNs form the basis of colloidal drug delivery systems, which are biodegradable and capable of being stored for at least one year. Chitosan (CT) is a biopolymer that has been widely used in the pharmaceutical field since it exhibits a number of interesting properties with wide range of pharmaceutical applications. A chitosan-based transport system has been developed for overcoming the Blood brain barrier [14]. The mechanism of action of carbamazepine is stabilizes the inactivated state of sodium channels, meaning that fewer of these channels are available to subsequently open, making brain cells less excitable carbamazepine has also been shown to potentiate GABA receptors made up of α1, β2 and γ2 subunits [15]. The present work aims to combine the advantages of SLN with the biological properties of chitosan in improving the antiepileptic property of lipophilic drug like carbamazepine by formulating a modified release drug delivery system [16].

## Materials and methods

### Materials

Carbamazepine was purchased from Yarrow chemicals Ltd. (Mumbai, India). Phospholipon R 80 H was a gift sample from Lipoid (Ludwigshafen, Germany). CT from Central fisheries research Institute (Cochin). Tristearin was procured from TCI Chemicals (India) Pvt. Ltd. All other reagents used in this study were of analar grade.

### Method of preparation of SLN dispersion

#### Preparation of CBZ loaded SLNs and the process of optimization

The CT-CBZ SLNs were prepared by using solvent injection method using ethanol as organic solvent. Tristearin, solubilized CT phospholipon R 80 H and drug is dissolved in the ethanol in definite ratio and warmed to 70°C. To the phosphate buffer solution (pH 7.4) a definite amount of tween 80 is added to prepare aqueous phase and kept for stirring which is maintain at 70°C. The organic phase was added drop wise with stirring to the pre warmed aqueous solution with the help of hypodermic needle. The mixture was then sonicated for varying time to obtain nanoparticles. The optimum parameters i.e. tween 80 concentrations in definite ratio and maximum sonication time resulted in maximum entrapment efficiency and controlled release were used for the preparation of SLN using similar method [17,18]. Three formulations were prepared by using different concentrations of tween 80 and sonication time to determine the effect of surfactant and sonication time on the potency of the SLNs.

#### Characterization of prepared SLNs

##### Fourier Transform infrared (FTIR) spectroscopic analysis

The FTIR spectra of CBZ, tristearin, phospholipon 80 H, CT CBZ loaded SLNs and physical mixture of lipids and drug in 1:1 ratio were recorded using FTIR spectrophotometer in the range of 4000–650 cm-^1^[19].

#### Measurement of particle size, polydispersity index and zeta potential

Particle size distribution of CBZ loaded SLNs was determined by laser scanning technique using Malvern instrument after appropriate dilution with distilled water. The mean particle size, polydispersity index and zeta potential were calculated for each formulation maintained at 25°C and polydispersity index will measure the size distribution of nanoparticles population [20].

#### Scanning electron microscopy (SEM)

The SEM analysis of prepared SLN was performed for morphological studies. The formulations are poured in to circular aluminium stubs using double adhesive tape, and coated with gold in HUS -5 GB vacuum evaporator , and observed in Hitachi S-3000 N SEM at an acceleration voltage of 10 Kv and a magnification of 5000X [21].

#### Differential scanning calorimetry (DSC)

DSC analysis was performed in order to investigate the melting and recrystallization behaviour of crystalline materials like SLNs. The samples were sealed in aluminium pans and measurements were recorded using DSC instrument. The samples were heated from 25 to 200°C at a heating rate of 100°C /min under nitrogen atmosphere [22].

#### Total drug content

From the prepared SLN formulation 1 ml of suspension is dissolved in the 10 ml of pH7.4 phosphate buffer solution (PBS) and ethanol mixture. The amount of carbamazepine was determined using UV spectrophotometer at 285 nm. The placebo formulation prepared similarly to drug loaded SLN is used as blank. The total drug content was calculated.

#### Entrapment efficiency (EE)

The prepared SLN dispersion was centrifuged with 15000 rpm speed for 30 min at 0°C using REMI cooling centrifuge. The free drug content is calculated with using of equation, EE = {total drug content-free drug content/ total drug content} X 100 from the supernatant portion.

#### *In-vitro* diffusion studies

The modified Franz diffusion cell at 37°C which is fitted with a dialysis membrane having a molecular weight cut off 350 Da was used for study. The membrane was soaked in boiling distilled water for 12 hours before mounting to Franz diffusion cell. SLN dispersion 2 ml was placed in to the donor compartment and the 20 ml of PBS is used to fill receptor compartment. With one hour interval 1 ml of sample was withdrawn and analysed using UV spectrophotometer at 285 nm [23].

### Assessment of antiepileptic activity

#### Experimental animals

Male albino rats of Wistar strain weighing 150 – 200 g were used for the study. They were housed in polypropylene cages and maintained under standard laboratory conditions with a 12–12 h light–dark cycle and as well as free access to standard rat pellet diet (Lipton, India Ltd.) and drinking water. They were acclimatized to laboratory conditions for 10 days before starting the experiment. The experimental protocol was approved by the institutional animal ethical committee (ref. no: 930/a/06/CPCSEA**).**

#### Maximal electro shock (MES) method

Maximal electroshock seizures were induced by an electroconvulsiometer (INCO, Ambala, India). The electrical stimulus (150 mA, 0.2 sec duration) was applied through ear electrodes. On giving the shock in normal animals, it should have a profile of tonic flexion, extension followed by clonus. Animals were selected by giving the shock 24 hours before the day of experiment. Animals that have shown all the three phases of convulsions were selected for the study. Eighteen rats were divided randomly into 3 groups (n = 6). Group I animals (control) were received 1 ml of 1% tween80/100mg body weight. Group II, standard reference group, received 1 mg/100 mg of 1% tween80/100mg, while Group III, test group received 1 ml of aqueous dispersion of CBZ SLN/100 mg body weight. The post extension phase in standard and test has greatly reduced. From The ANOVA analysis it is shown that test and standard groups has shown faster recovery compared to control. The assessment of the extension/flexion (E/F) ratio in MES model has been documented as a major end point used to assess the antiepileptic potentials of drugs effective in generalized tonic–clonic (grandmal) seizures. Pure CBZ is used as standard drug in MES model, which primarily act by blocking voltage-dependent Na + channels. The experiments were conducted one hour after oral administration.

#### Isoniazid (INH) induced convulsions

The animals were divided in to three groups of 6 each. Group I was maintained as control and received 1% tween 80, Group II was maintained as standard received pure CBZ and Group III were received CBZ optimised SLN formulation. One hour after administration of drug dose INH at dose of 300 mg was administered, the rats were kept in isolated chamber during the next 120 min the occurrence of clonic seizures, tonic seizures and death is recorded. The percentage of seizures or death occurring in the control group taken as 100%.The suppression of these effects in the treated groups is calculated as percentage of controls.

## Results and discussion

SLNs were prepared by solvent injection technique which relies on the rapid diffusion of solvent across the solvent–lipid interface with aqueous phase; hence the rate of diffusion of organic solvent through the interface seems to be critical parameter for particle size determination. In the present work ethanol was selected as miscible solvent due to its solubilising potential for tristearin, and phospholipon. The smaller particle size is achieved due to addition of tween 80 and phospholipon in the organic phase.

### FT-IR spectroscopy

**Figure 1:**
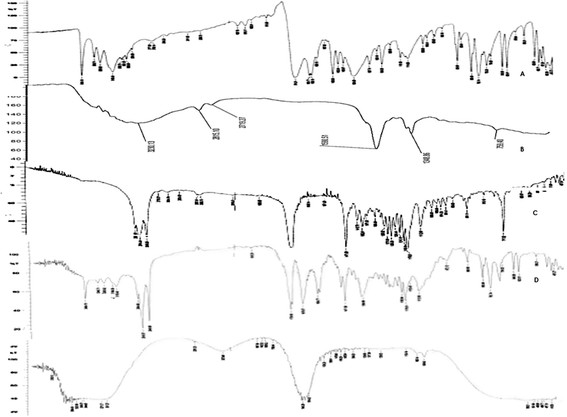
FTIR spectra CBZ (A), Chitosan (B) Tristearin (C), Physical mixture of CBZ and Tristearin (D) and CBZ SLN (E).

FT-IR spectroscopy was employed to obtain conformational information about the lipid molecules and it is used to investigate the interactions between lipid, drug and other excipients. From the FT-IR spectra of pure drug, optimised formulation and physical mixture it is confirmed that there are no particular interactions between the lipids and drug (Figure 1). Interpretation of FT-IR spectra of CBZ SLN was done the functional peaks are tabulated in Table 1. The presence of characteristic peaks of CBZ physical mixture as well as formulation reveals that the drug remains intact in the formulation without any interactions.

**Table 1:** FTIR interpretation of CBZ SLN comparative to pure drug physical mixture and polymers

**Functional group**	**Drug**	**SLN formulation**	**Physical mixture**	**Tristearin**	**Phospholipon 80 H**
N-H stretch	3464 cm^-1^	3444.8 cm^-1^	3464.15 cm^-1^	Absent	Absent
C = 0 stretch	1677 cm^-1^	1635.64 cm^-1^	1678.07 cm^-1^	Absent	Absent
Aromatic C-H stretch	3080.75 cm^-1^	Absent	2954.95 cm^-1^	2954.95 cm^-1^	2954.95 cm^-1^
C = C stretch	1605 cm^-1^	1635.64,1558.48 cm^-1^	1604.77 cm^-1^	Absent	Absent
C≡N	1384.89 cm^-1^	1373.32 cm^-1^	1384.89 cm^-1^	Absent	Absent
CHO aldehydic group	Absent	1732.80 cm^-1^	1735.93 cm^-1^	1728.22 cm^-1^	1725.92 cm^-1^
Methylene C-H asym stretch	Absent	Absent	2916.37 cm^-1^	2914.44 cm^-1^	2916.37 cm^-1^
Polymeric	Absent	3444.87 cm^-1^	3280.92 cm^-1^	Absent	3398.57 cm^-1^
					3377.26 cm^-1^
O-H stretch		3419.97 cm^-1^			3281.93 cm^-1^

### Differential scanning calorimetry (DSC)

**Figure 2:**
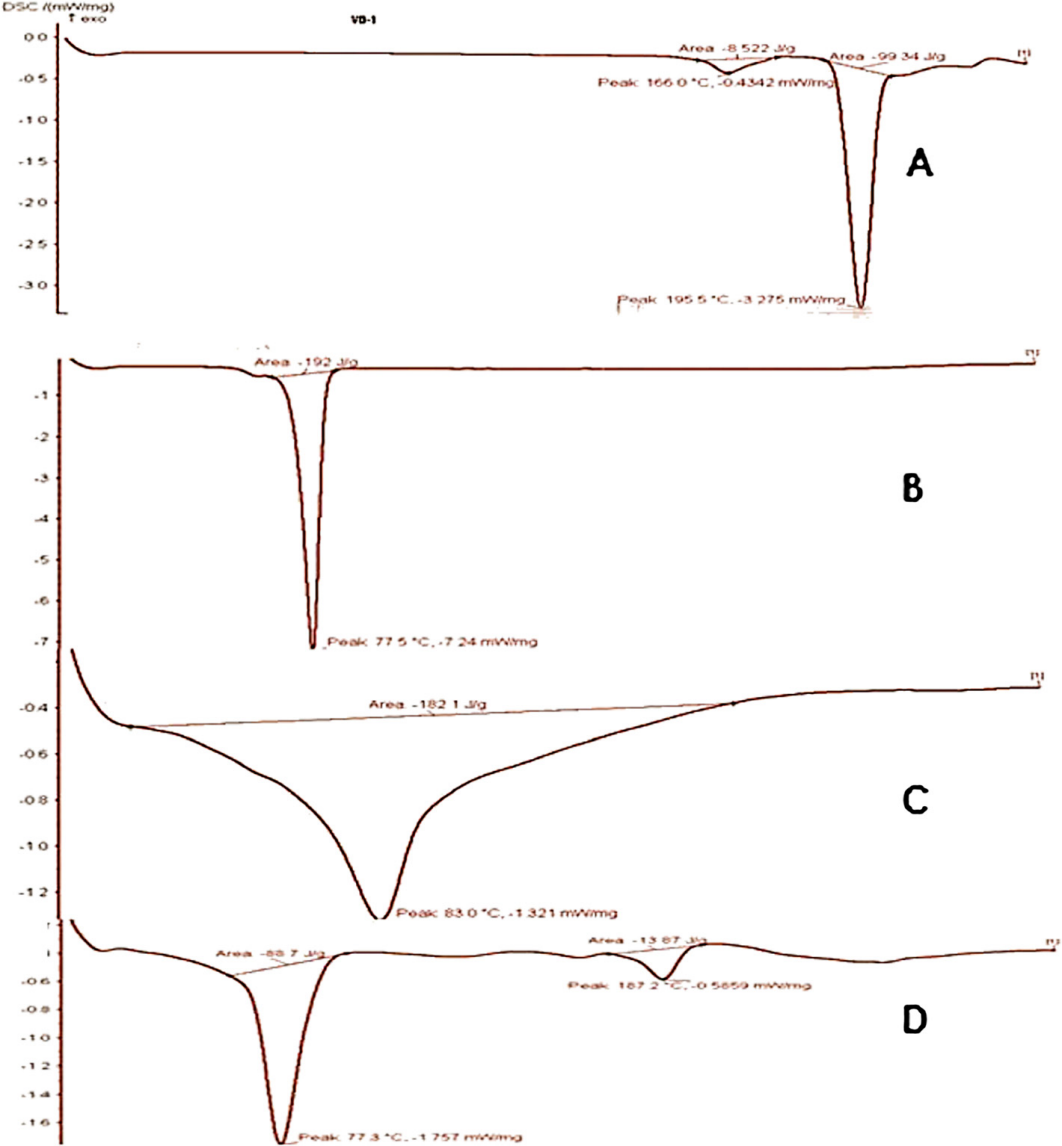
DSC thermograms of CBZ (A), Tristearin (B), Physical mixture of CBZ and Tristearin (C) and CBZ SLN D.

In the development of SLNs the confirmation of desired physical state of matrix lipid is of crucial importance which can be determined by the DSC. When the DSC thermograms of the bulk lipids and corresponding SLNs are compared the difference in the position and shape of the signals are usually observed. Figure 2 shows DSC curves of CBZ tristearin (TS), physical mixture and CBZ-SLN. The physical mixture DSC thermogram of CBZ indicated that there were no interaction between the drugs and excipients which can be accessed from the peaks in Figure 2. The DSC thermogram of CBZ SLN did not show melting point for CBZ which is 172°C, which is close to the value reported in literature hence the procured drugs are in pure state. CBZ SLNs did not show the melting peak for the CBZ around 172°C. This shows that CBZ was not in crystalline state but it is in amorphous state.

### SEM

**Figure 3:**
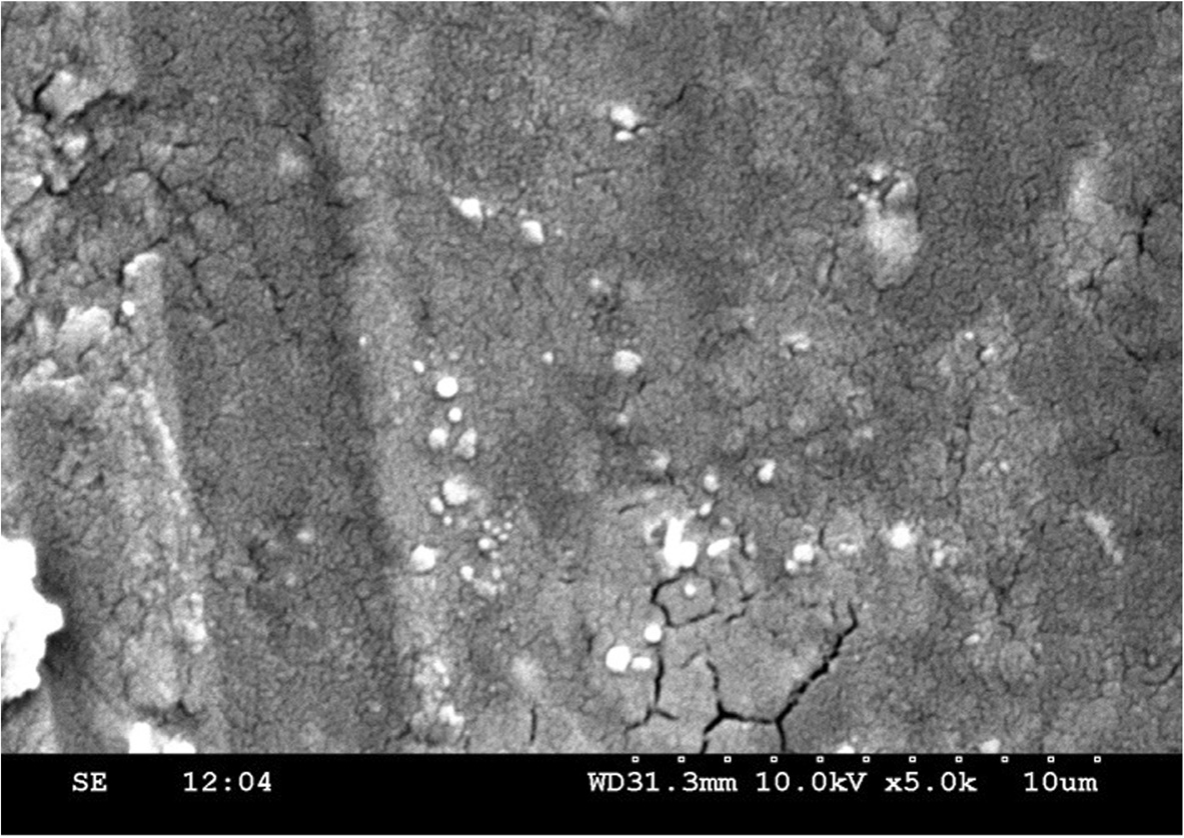
CBZ loaded SLN SEM photograph.

The SEM photograph (Figure 3) of optimised formulation reveals that SLNs were spherical and moderately uniform.

### Entrapment efficiency of CBZ CT SLNs

The EE of the fabricated batches was in the range of 48.84% to 66.7% the total drug content and free dug content of the 3 formulations are shown in the following Table 2.

**Table 2:** Free drug content, Total drug content and %EE of SLN formulationsange

**S. No.**	**Formulation**	**Free drug content (mg)**	**Total drug content (mg)**	**%EE**
1	F1	10.2	20.136	48.84
2	F2	23.4	34.86	32.87
3	F3	8.6	25.8	66.7

### Particle size determination

**Figure 4:**
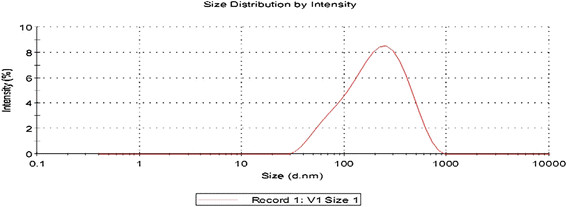
CBZ SLN F1 Size distribution chart.

Figure 4 showed, narrow distribution width and considerable narrow particle size for SLNs prepared by solvent injection method and confirming the good dispersion quality. The measurement of zeta potential allows for prediction about the storage stability of colloidal particles, as the particle aggregation will be less to the charged particles. For the prepared SLNs the Zeta Potential (mV): particle size and polydispersity index are tabulated in Table 3.

**Table 3:** Zeta potential ,particle size and polydispersity index of SLN Formulations

**Formulation**	**Size average (d.nm):**	**Polydispersity index**	**Zeta potential**
F1	168.7 ± 1.8 nm	0.206	−28.9 ± 2.0 mV
F2	187.5 ± 2.8 nm	0.219	−27.60 ± 1.8 mV
F3	154.0 ± 1.5 nm	0.309	−32.12 ± 1.4 mV

### *In-vitro* diffusion studies

**Figure 5:**
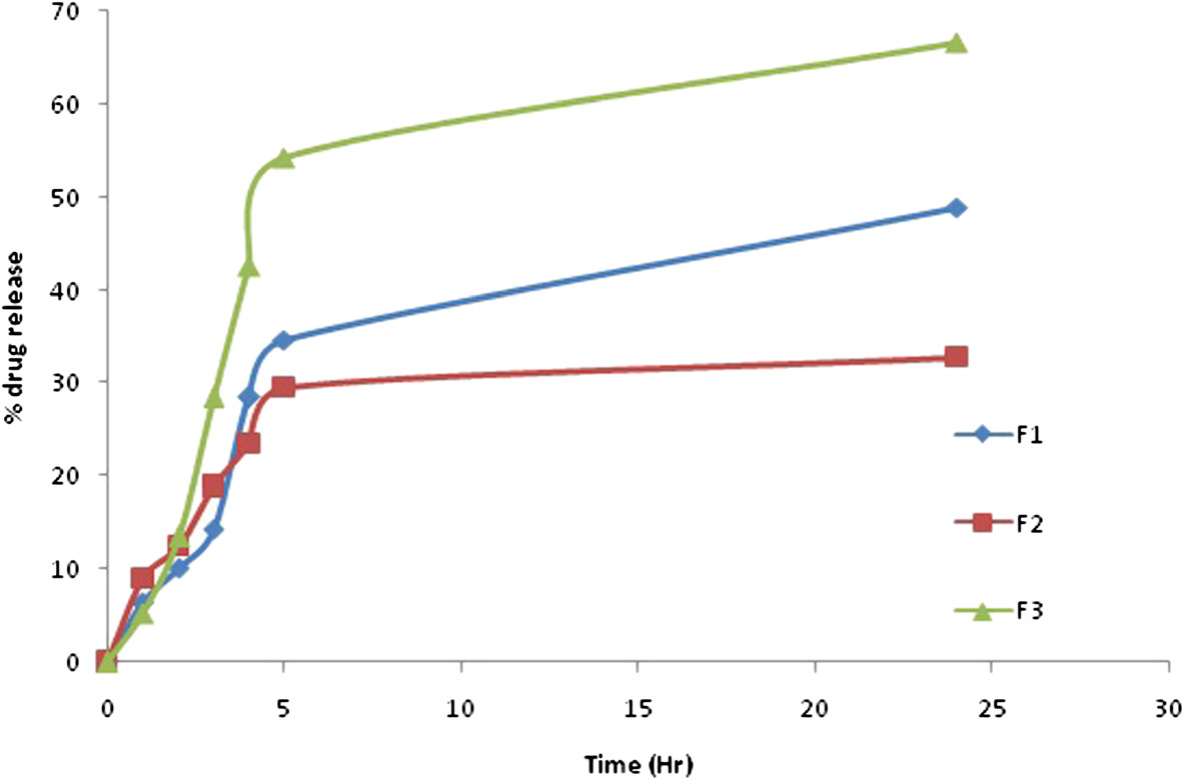
Comparative release profile of CBZ SLN in 7.4 PBS for F1-F3.

Modified Franz diffusion cells with dialysis membrane were used in our study. This dialysis membrane allowed the transfer of drug immediately in to receiver compartment. The % drug release of CBZ from different formulation of SLNs is depicted in Figure 5. The formulation F3 showed 66.7% of drug was released from SLNs in 24 hours.

### Antiepileptic activity

#### MES method

**Figure 6:**
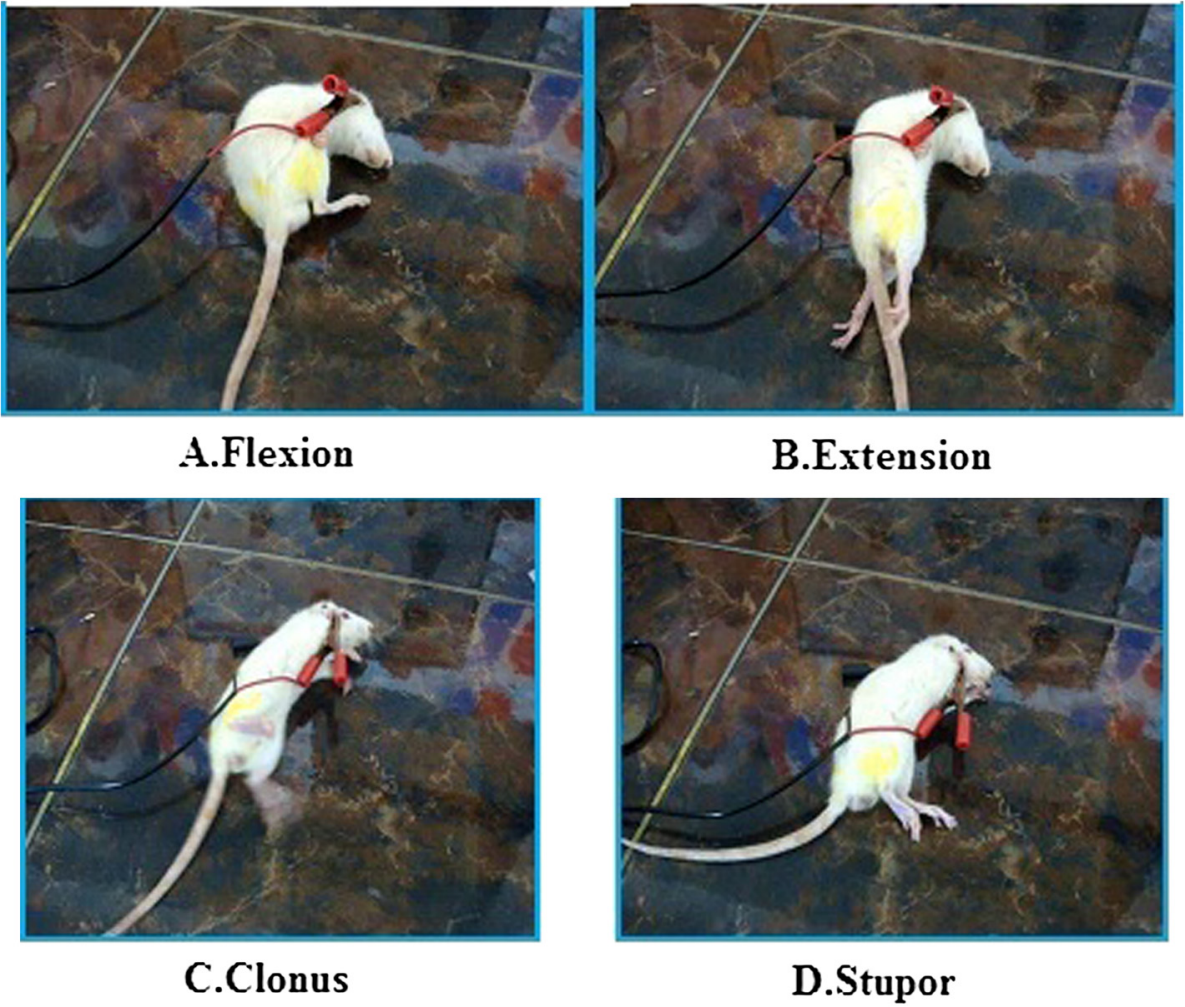
The various stages in MES induced seizures.

In this model, CBZ-CT SLNs have shown better activity compared to standard and control (Figure 6). The time for onset of convulsions in CBZ SLN s treated group took more time compared to test and standard**.** Reported in Table 4.

**Table 4:** Effect of a single dose administration of CBZ SLN on reducing the duration of epileptic phases in Maximal Electric Shock (MES) model

**S. No.**	**Phases in MES model**	**Control (Mean ± SEM) (Sec)**	**Standard (Mean ± SEM) (Sec)**	**Test (Mean ± SEM) (Sec)**	**Significance**
1	Flexion	1.167 ± 0.1667	Nil	Nil	(P<0.05) significant
2	Extension	26.50 ± 1.607	0.0 ± 0	0.0 ± 0	(P<0.05) significant
3	Clonus	18.83 ± 0.7923	15.83 ± 2.787	4.667 ± 1.211	(P<0.05) significant
4	Stupor	72 ± 2.828	35.67 ± 6.377	24.33 ± 7.528	(P<0.05) significant
5	Recovery	Recovered	Recovered	Recovered	significant

### INH method

In this model, CBZ SLNs has shown better activity compared to standard and control. The time for onset of convulsions in the CBZ SLNs has taken more time compared to test and standard. Reported in Table 5.

**Table 5:** Effect of a single dose administration of CBZ SLN on reducing the duration of epileptic phases in INH indused epileptic model

**INH Induced method**	**Control (Mean ± SEM) (Min)**	**Standard (Mean ± SEM) (Min)**	**Test (Mean ± SEM) (Min)**	**Significance**
Time for onset of convulsions	28 ± 1.195	58.17 ± 6.676	135.0 ± 25.10	(P<0.05) significant

## Conclusion

In the present work CBZ a lipophilic drug when incorporated in to Chitosan SLN shows controlled release of the drug from formulations. The results obtained in this work suggest that SLN with chitosan have a great potential for delivery of CBZ due to their high encapsulation efficiency, high physical stability. The combination of chitosan with SLN can improve the SLNs properties as carrier for CBZ because the SLN-chitosan-CBZ exhibited an improved anti epileptic activity when compared against CBZ in the treatment of seizures.

## Competing interests

No disclosures. There is no affiliation, financial agreement or any other involvement with any company.

## Authors’ contributions

RN carried out the formulation, in vitro characterization and drafted the manuscript. CY carried out the in vivo studies. VP coordinated for the study. PY designed the protocol of the study. AK participated in design and coordination of the study. All authors read and approved the final manuscript.
